# Circular RNA circ_0026359 Enhances Cisplatin Resistance in Gastric Cancer via Targeting miR-1200/POLD4 Pathway

**DOI:** 10.1155/2020/5103272

**Published:** 2020-08-13

**Authors:** Zongyao Zhang, Xin Yu, Bo Zhou, Jiajia Zhang, Jiacong Chang

**Affiliations:** Department of General Surgery, The First Affiliated Hospital of Anhui Medical University, Hefei, Anhui, China

## Abstract

Human gastric cancer is one of the most common malignant tumors with a poor prognosis. Cisplatin (CDDP) is a well-known first-line chemotherapeutic drug. Acquired resistance retards the clinical application of CDDP in gastric cancer. In this study, circular RNA circ_0026359 was demonstrated to be overexpressed in gastric cancer tissues/cells compared with normal gastric tissues/cells and was overexpressed in CDDP-resistant gastric cancer tissues/cells compared with CDDP-sensitive gastric cancer tissues/cells. High levels of circ_0026359 were associated with low overall survival (OS) and relapse-free survival (RFS) rates in gastric cancer patients. circ_0026359 was examined to promote CDDP resistance in gastric cancer cells. circ_0026359 directly interacted and negatively regulated miR-1200. POLD4 was a direct target of miR-1200. miR-1200/POLD4 pathway mediated the promoting role of circ_0026359 in CDDP resistance of gastric cancer. circ_0026359 could be used as a potential target for CDDP-resistant gastric cancer therapy.

## 1. Introduction

Human gastric cancer is one of the most common malignant tumors, and its incidence rate and mortality remain high worldwide [1, 2]. For advanced gastric cancer patients, their survival time is less than 1 year [3, 4]. Cisplatin (CDDP) is a well-known first-line chemotherapeutic drug which is usually used in combination with other chemotherapy drugs for gastric cancer [5, 6]. Acquired resistance retards the clinical application of CDDP in gastric cancer [5, 7]. Many mechanisms were reported to be involved in CDDP resistance of human gastric cancer, such as miR-101/VEGF-C pathway [8], miR-34a/PI3K/AKT/survivin pathway [9], miR-1271/IGF1R, IRS1, mTOR, BCL2 pathway [10], and circ-FN1/miR-182-5p pathway [7]. It is desirable for further study of the intrinsic and detailed mechanisms in CDDP resistance of human gastric cancer.

Circular RNA (circRNA) is one type of noncoding RNAs, which was identified to play important roles in many physiological processes lately [11, 12]. circRNAs are produced through 5′ and 3′ end linking to form closed loops and usually act as sponges of miRNAs to regulate downstream genes [11, 13]. Recently, circRNAs were reported to play important roles in nearly all kinds of human cancers. For example, circ_0020397 was examined to regulate cell viability, apoptosis, and invasion via miR-138/TERT, PD-L1 pathway in human colorectal cancer [14]; circ-VPS13C-has-circ_001567 was documented to promote cell proliferation and invasion in ovarian cancer cells [15]; circ-ABCB10 was reported to promote cell proliferation and progression of human breast cancer by sponging miR-1271 [16]; circ_0046264 suppressed lung cancer via miR-1245/BRCA2 pathway [17]; and circ-PVRL3 suppressed proliferation and migration of gastric cancer cells [18]. Moreover, circ-AKT3, circ_0081143, and circ-FN1 were documented to enhance CDDP resistance of human gastric cancer cells [7, 19, 20]. Specifically, it is reported that circ_0026359 was significantly upregulated in gastric cancer tissues compared to the normal ones [21]. However, the exact role of circ_0026359 in human gastric cancer and in CDDP resistance of gastric cancer cells remains unclear.

In the present study, we have examined that the expression levels of circ_0026359 were higher in human gastric cancer tissues/cells compared with normal gastric tissues/cells and were higher in CDDP-resistant gastric cancer tissues/cells compared with CDDP-sensitive gastric cancer tissues/cells. High levels of circ_0026359 were associated with both low overall survival (OS) rates and relapse-free survival (RFS) rates in gastric cancer patients. Depletion of circ_0026359 decreased the resistance of CDDP-resistant gastric cancer cells to CDDP. miR-1200 was examined to be directly interacted with circ_0026359 and was negatively regulated by circ_0026359. POLD4 was determined to be a direct target of miR-1200. Rescue experiments had examined that miR-1200/POLD4 mediated the role of circ_0026359 in CDDP resistance of gastric cancer cells. Therefore, circ_0026359 was an important noncoding RNA contributing to CDDP resistance of human gastric cancer. circ_0026359 could be used as a potential target for adjuvant therapy in CDDP-resistant gastric cancer patients.

## 2. Materials and Methods

### 2.1. Gastric Cancer Patients and Tissue Samples

In this study, we collected 55 pairs of fresh gastric cancer tissues and normal gastric tissues from 55 gastric cancer patients in the First Affiliated Hospital of Anhui Medical University (Hefei, Anhui, China) during 2013-2015. In these 55 gastric cancer patients, 22 of them received CDDP-based chemotherapy and had got CDDP resistance, and the other 33 patients did not receive CDDP based-chemotherapy and they were CDDP sensitive. These 55 patients were followed up for more than 5 years, and their OS and RFS rates were collected. We had got local approval from the Institutional Review Boards of Anhui Medical University before work. This study was performed according to the Code of Ethics of the World Medical Association (Declaration of Helsinki). Informed consents were signed by these 55 gastric cancer patients.

### 2.2. Cell Culture

Normal gastric cell line GES-1 and gastric cancer cell lines HGC-27, AGS, BGC-823, SGC-7901, and MKN-45 were used in this study. These cells were got from the ATCC (American Type Culture Collection) (Rockville, MD). We developed CDDP-resistant cell lines SGC-7901/CDDP and MKN-45/CDDP. Briefly, SGC-7901 and MKN-45 cells were treated with 1 *μ*g/ml CDDP continuously for at least 6 months; the resistance of SGC-7901/CDDP and MKN-45/CDDP cells to CDDP was evaluated by a MTT assay. As recommended, these cells were maintained in 37°C, 5% CO_2_, and humidified cell incubators.

### 2.3. RT-qPCR (RT-Quantitative PCR)

In this study, RT-qPCR was carried out to examine expression levels of circRNAs, miRNAs, and mRNAs by using SYBR Green qPCR SuperMix (Thermo Fisher Scientific) as described in former publication [22]. The 2^-*ΔΔ*Ct^ method was used for quantitative analysis. GAPDH and U6 were detected as the control, respectively. The primers used were: circ_0026359 F, 5′-AATGAGACGGAGTTGACAGA-3′ and R, 5′-CACGAGCATCCTTGAGC-3′; POLD4 F, 5′-TGGGCACAGCAAGG-3′ and R, 5′-CAGGCCAGGTCAAACT-3′; GAPDH F, 5′-AGAAGGCTGGGGCTCATTT G-3′ and R, 5′-AGGGGCCATCCACAGTCTTC-3′; miR-1200 F, 5′-ACACT CCAGCTGGGCTCCTGAGCCATTCTG-3 and R, 5′-CTCAACTGGTGTCGTGGAGT CGGCAATTCAGTTGAGGAGGCTCA-3′; and U6 F, 5′-CTCGCTTCGGCAGCACA-3′ and R, 5′-AACGCTTCACGAATTTGCGT-3′.

### 2.4. RNA Oligonucleotides and Transfection

RNA oligonucleotides used in this study including circ_0026359-siRNAs (si-circ_0026359#1 and si-circ_0026359#2), negative control siRNA (si-NC), miR-1200/miR-NC (negative control) mimics, and miR-1200-inhibitor/negative control- (NC-) inhibitor were synthesized in GenePharma (Shanghai, China). RNA oligonucleotide transfection was carried out using Lipofectamine™ 3000 (Thermo Fisher Scientific) as described in previous study [22].

### 2.5. Plasmid Construction and Transfection

Wild-type circ_0026359 sequence, mutant circ_0026359 sequence, wild-type human POLD4 3′UTR sequence, and mutant POLD4 3′UTR sequence were cloned into luciferase reporter plasmid pmirGLO (Promega, China) for luciferase reporter assay analysis, and they were designated as hsa_circ_0026359-wt, hsa_circ_0026359-mt, hsa_POLD4 3′UTR-wt, and hsa_POLD4 3′UTR-mt, respectively. circRNA/miRNA and plasmids/miRNA cotransfection was performed using Lipofectamine™ 3000 (Thermo Fisher Scientific) as recommended.

### 2.6. Cell Functional Assays

In this study, the MTT assay was carried out to evaluate cell viabilities essentially as described in former study [23]. Briefly, cells were harvested 24 hours after transfection and seeded into 96-well plates (3 × 10^4^ per well), cells were maintained for 24 hours and then treated with 1 *μ*g/ml CDDP, and cell viabilities were tested 24, 36, and 48 hours later.

IC50 analysis was carried out to examine cell sensitivity to CDDP as described in previous study [23]. Briefly, cells were treated with 0.25, 0.5, 1, 2, 4, 8 *μ*g/ml CDDP for 36 hours, and MTT examination was performed. Cell growth curves were made, and cell IC50 values for CDDP were analyzed.

For cell colony formation, 1000 cells per well were seeded into 6-well plates and treated with 1 *μ*g/ml CDDP for 48 hours. Cell colony formation was examined 10 days later as described previously [23].

Caspase-3/7 activity assay was performed by using a Caspase-Glo 3/7 Assay kit (Promega) as described in previous study [24].

DNA fragmentation was examined to evaluate cell apoptosis which was carried out as described in former study [25].

For flow cytometry, cells were harvested, fixed, and incubated with Annexin V-FITC (Beyotime Institute of Biotechnology) and propidium iodide (Sigma-Aldrich; Merck KGaA) at room temperature for 30 min. Flow cytometric analysis was carried out to examine cell apoptosis.

### 2.7. Nuclear and Cytoplasmic circRNA Examination

Cell nuclear and cytoplasmic RNAs were isolated by using a PARIS™ Kit (Thermo Fisher Scientific), and the expression levels of circRNAs were examined by RT-qPCR as described in former study [22]. U6 and GAPDH were detected as the control.

### 2.8. Luciferase Reporter Assay

A Dual-Luciferase® Reporter Assay System (Promega) was used for luciferase reporter assay examination which was performed essentially as described earlier [22].

### 2.9. RNA Pull-Down Assay

The RNA pull-down assay was performed to evaluate the interaction between circ_0026359 and miR-1200 as described previously [22]. Biotinylated circ_0026359 probe was used, and circ_0026359/miR-1200 in the extracts were examined by RT-qPCR.

### 2.10. Western Blot

Protein levels of POLD4 were examined by western blot, which was performed according to former study [22]. PLOD4 Rabbit Polyclonal antibody (1 : 1000, 26209-1-AP, Proteintech Group, Inc., Chicago, USA) and *α*-tubulin Mouse Monoclonal antibody (1 : 10000, 66031-1-Ig, Proteintech Group, Inc., Chicago, USA) were used. *α*-Tubulin was examined as the control.

### 2.11. Statistical Analyses

For each experiment, at least 3 independent repeats were carried out, and the results in figures represented the average. A two-tailed *t*-test was performed for statistical analysis in RT-qPCR, MTT assay, IC50 analysis, cell colony formation assay, caspase-3/7 activity assay, DNA fragmentation assay, luciferase reporter assay, and RNA pull-down assay. OS and RFS analysis was carried out using Kaplan-Meier curves, and a log-rank test was performed for statistical analysis. *P* < 0.05 was considered to be statistically significant.

## 3. Results

### 3.1. circ_0026359 Was Overexpressed in Gastric Cancer and Associated with CDDP Resistance and Poor Survival Rates in Gastric Cancer Patients

**Figure 1:**
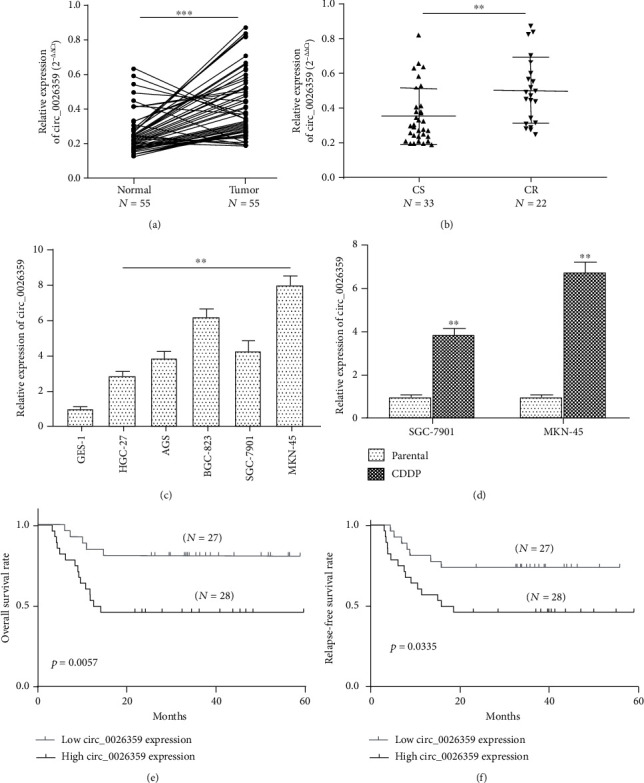
circ_0026359 was overexpressed in gastric cancer and associated with CDDP resistance and poor survival rates in gastric cancer patients. (a) Expression levels of circ_0026359 in 55 pairs of gastric cancer tissues and normal gastric tissues were examined by RT-qPCR. (b) Expression levels of circ_0026359 in 33 CDDP-sensitive (CS) and 22 CDDP-resistant (CT) gastric cancer tissues. (c) Expression levels of circ_0026359 in normal gastric cell GES-1 and gastric cancer cells HGC-27, AGS, BGC-823, SGC-7901, and MKN-45 were examined by RT-qPCR. (d) Expression levels of circ_0026359 in CDDP-resistant gastric cancer cells SGC-7901/CDDP and MKN-45/CDDP and parental gastric cells SGC-7901 and MKN-45. GAPDH was examined as control for RT-qPCR. (e) Overall survival (OS) rates and (f) relapse-free survival (RFS) rates in gastric cancer patients with high circ_0026359 expression and low circ_0026359 expression were analyzed by Kaplan-Meier curves. ^∗∗^*P* < 0.01; ^∗∗∗^*P* < 0.001.

In this study, 55 fresh gastric cancer tissues and 55 normal gastric tissues were collected, and the expression levels of circ_0026359 were examined by RT-qPCR. As shown in Figure 1(a), the expression levels of circ_0026359 were much higher in gastric cancer tissues compared with normal gastric tissues. In these 55 gastric cancer samples, 33 of them were CDDP sensitive and the other 22 were CDDP resistant. As shown in Figure 1(b), the expression levels of circ_0026359 were much higher in CDDP-resistant gastric cancer tissues compared with CDDP-sensitive gastric cancer tissues. To confirm this result, the expression levels of circ_0026359 in gastric cancer cell lines and normal gastric cell lines were examined. Compared with normal gastric cell GES-1, the expression levels of circ_0026359 were extremely higher in gastric cancer cells HGC-27, AGS, BGC-823, SGC-7901, and MKN-45 (Figure 1(c)). For further study, CDDP-resistant gastric cells SGC-7901/CDDP and MKN-45/CDDP were developed. As shown in Figure 1(d), the expression levels of circ_0026359 were much higher in SGC-7901/CDDP and MKN-45/CDDP cells compared with their parental SGC-7901 and MKN-45 cells, respectively. Therefore, circ_0026359 was overexpressed in gastric cancer tissues/cells compared with normal gastric tissues/cells; circ_0026359 was overexpressed in CDDP-resistant gastric cancer tissues/cells compared with CDDP-sensitive gastric cancer tissues/cells.

For further study, these 55 gastric cancer patients were followed up for at least 5 years, and the correlations between their OS/RFS rates and circ_0026359 expression were analyzed by Kaplan-Meier curves. As shown in Figures 1(e) and 1(f), gastric cancer patients with high circ_0026359 expression exhibited both lower OS rates (*P* = 0.0057) and RFS rates (*P* = 0.0335) compared with patients with low circ_0026359 expression. Therefore, high levels of circ_0026359 were associated with poor survival rates in gastric cancer patients.

### 3.2. Depletion of circ_0026359 Decreased CDDP Resistance of Gastric Cancer Cells

**Figure 2:**
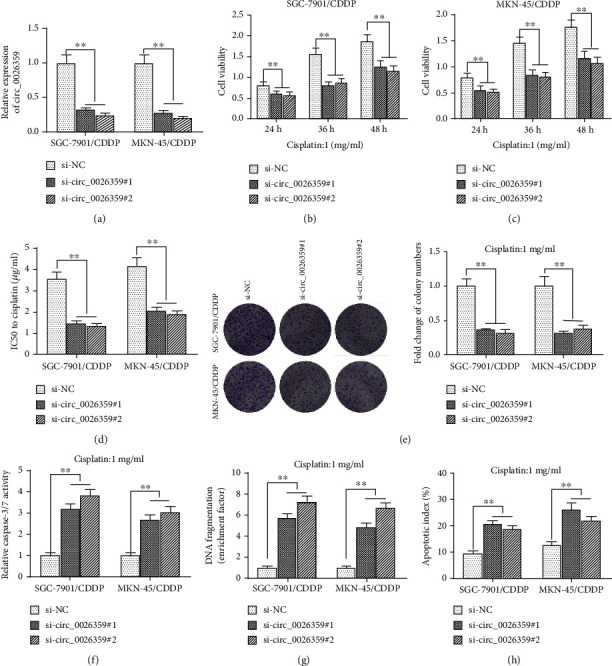
Depletion of circ_0026359 decreased CDDP resistance of gastric cancer cells. CDDP-resistant gastric cancer cells SGC-7901/CDDP and MKN-45/CDDP were transfected with si-circ_0026359#1, si-circ_0026359#2, or siNC. (a) Expression levels of circ_0026359 were examined by RT-qPCR. GAPDH was used as control. (b, c) These cells were treated with 1 *μ*g/ml CDDP for 24, 36, and 48 hours, and cell viabilities were examined by the MTT assay. (d) IC50 for CDDP in these cells were analyzed by the MTT assay. (e) Cell colony formation assay was performed in these cells with 1 *μ*g/ml CDDP treated for 48 hours. (f) Caspase-3/7 assay, (g) DNA fragmentation assay, and (h) flow cytometry with Annexin V-FITC and PI staining were performed in these cells with 1 *μ*g/ml CDDP treated for 48 hours. ^∗∗^*P* < 0.01.

siRNAs were introduced for circ_0026359 depletion. As shown in Figure 2(a), circ_0026359-siRNA (indicated as si-circ_0026359#1 and si-circ_0026359#2) significantly decreased circ_0026359 levels in CDDP-resistant cells SGC-7901/CDDP and MKN-45/CDDP compared with negative control siRNA (indicated as si-NC). As determined by the MTT assay, on exposure to 1 *μ*g/ml CDDP, cell viabilities of SGC-7901/CDDP and MKN-45/CDDP cells after transfection with si-circ_0026359#1 or si-circ_0026359#2 decreased dramatically compared with negative control cells (cells transfected with si-NC), respectively, at 24 h, 36 h, and 48 h (Figures 2(b) and 2(c)). In SGC-7901/CDDP and MKN-45/CDDP cells, depletion of circ_0026359 by si-circ_0026359#1 or si-circ_0026359#2 also extremely decreased the IC50 for CDDP (Figure 2(d)). Moreover, knockdown of circ_0026359 significantly decreased cell colony formation of SGC-7901/CDDP and MKN-45/CDDP cells on exposure to 1 *μ*g/ml CDDP (Figure 2(e)). In addition, depletion of circ_0026359 also increased cell apoptosis of SGC-7901/CDDP and MKN-45/CDDP cells treated with 1 *μ*g/ml CDDP as determined by caspase-3/7 activity (Figure 2(f)), DNA fragmentation (Figure 2(g)), and flow cytometry (Figure 2(h)). Therefore, depletion of circ_0026359 with siRNAs decreased CDDP resistance in gastric cancer cells.

### 3.3. miR-1200 Directly Bound to circ_0026359

**Figure 3:**
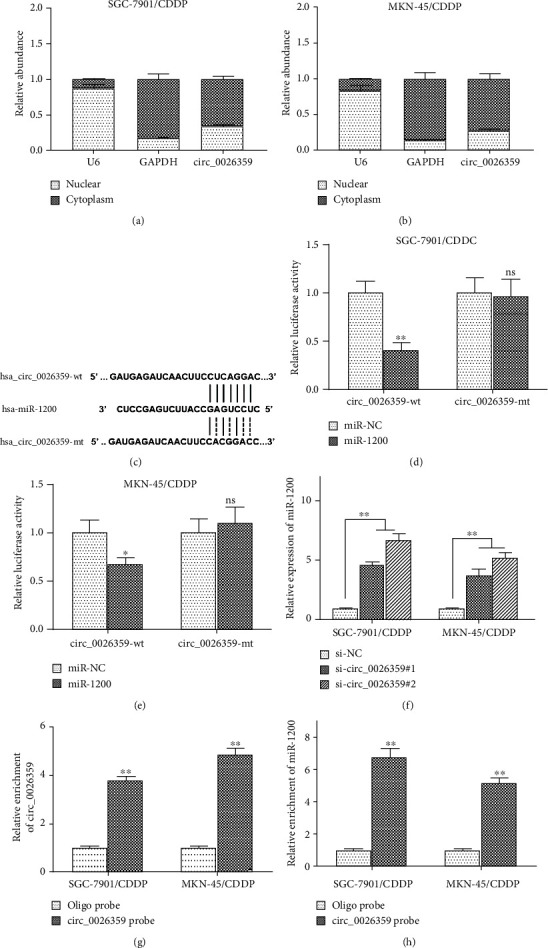
miR-1200 directly bound to circ_0026359. (a, b) Location of circ_0026359 in SGC-7901/CDDP and MKN-45/CDDP cells was examined by nuclear and cytoplasmic separation assay. circ_0026359 was detected by RT-qPCR. U6 and GAPDH were examined as control. (c) Predicted consequential pairing of circ_0026359 with miR-1200 and the mutant consequence of circ_0026359 used in luciferase reporter assay. (d, e) Luciferase reporter assay of SGC-7901/CDDP and MKN-45/CDDP cells cotransfected with miR-1200 mimics/negative control miRNA (miR-NC) and luciferase reporter plasmid pmirGLO containing wild-type circ_0026359 sequence (hsa_circ_0026359-wt)/mutant circ_0026359 sequence (hsa_circ_0026359-mt). (f) RT-qPCR was carried out to examine miR-1200 levels in SGC-7901/CDDP and MKN-45/CDDP cells after transfection with si-circ_0026359#1, si-circ_0026359#2, or siNC. U6 was detected as control. (g, h) RNA pull-down assay: (g) circ_0026359 and (h) miR-1200 were enriched by biotinylated circ_0026359 probe, and their levels were examined by RT-qPCR. ^∗^*P* < 0.05; ^∗∗^*P* < 0.01. ns: no significance.

For further study, the exact mechanisms of circ_0026359 involved in CDDP resistance of gastric cancer cells were explored. We firstly examined the localization of circ_0026359. As shown in Figures 3(a) and 3(b), circ_0026359 is mainly located in the cytoplasm of SGC-7901/CDDP and MKN-45/CDDP cells. As known, circRNAs usually acted as miRNA sponges in cell cytoplasm. We next searched potential target miRNAs that might be directly interacted with circ_0026359 using software. There were direct binding sites between miR-1200 and circ_002635 (Figure 3(c)). As determined by the luciferase reporter assay, when cotransfected with luciferase reporter plasmid containing wild-type circ_0026359 (wt-circ_0026359) and miR-1200 mimics in SGC-7901/CDDP and MKN-45/CDDP cells, the luciferase reporter activities were significantly suppressed compared with transfected with wt-circ_0026359 plasmid and miR-NC mimics, respectively; however, there were no significant changes of the luciferase reporter activities between cotransfection with mutant circ_0026359 (mt-circ_0026359) plasmid/miR-1200 mimics and mt-circ_0026359 plasmid/miR-NC mimics (Figures 3(d) and 3(e)). Moreover, si-circ_0026359#1 and si-circ_0026359#2 dramatically increased the miR-1200 levels in both SGC-7901/CDDP and MKN-45/CDDP cells compared with si-NC control (Figure 3(f)). Furthermore, as determined by the RNA pull-down assay, circ_0026359 probe enriched both circ_0026359 (Figure 3(g)) and miR-1200 (Figure 3(h)) in SGC-7901/CDDP and MKN-45/CDDP cells. Therefore, miR-1200 directly bound to circ_0026359 in CDDP-resistant gastric cancer cells.

### 3.4. POLD4 Was a Direct Target of miR-1200

**Figure 4:**
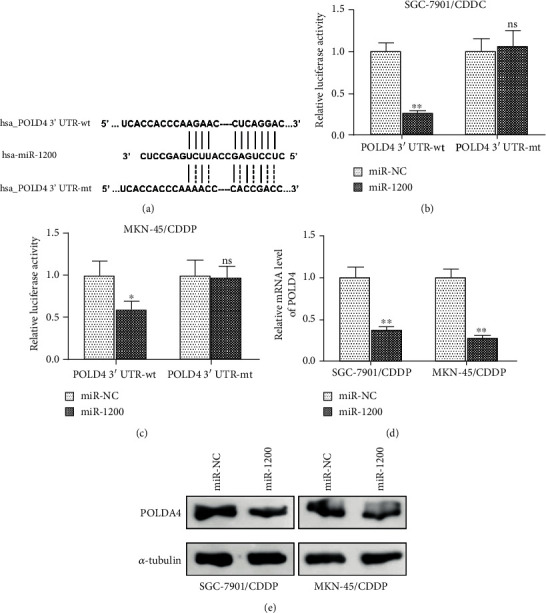
POLD4 was a direct target of miR-1200. (a) Predicted consequential pairing of miR-1200 with the POLD4 3′UTR and the mutant consequence of POLD4 3′UTR used in the luciferase reporter assay. (b, c) Luciferase reporter assay of SGC-7901/CDDP and MKN-45/CDDP cells cotransfected with miR-1200/miR-NC mimics and luciferase reporter plasmid pmirGLO containing wild-type POLD4 3′UTR sequence (hsa_POLD4 3′UTR-wt)/mutant POLD4 3′UTR sequence (hsa_POLD4 3′UTR-mt). (d) mRNA levels of POLD4 in SGC-7901/CDDP and MKN-45/CDDP cells after transfection with miR-1200/miR-NC mimics were examined by RT-qPCR. U6 was detected as control. (e) Protein levels of POLD4 in SGC-7901/CDDP and MKN-45/CDDP cells after transfection with miR-1200/miR-NC mimics were examined by western blot. *α*-Tubulin was detected as control. ^∗^*P* < 0.05; ^∗∗^*P* < 0.01. ns: no significance.

Next, potential targets of miR-1200 were predicted by online software TargetScan. POLD4 was found to be a candidate target of miR-1200, and the miR-1200-binding site with the 3′UTR of POLD4 mRNA was 5′-AGAA-3′ and 5′-CUCAGGA-3′ (Figure 4(a)). As determined by the luciferase reporter assay, when cotransfected with luciferase reporter plasmid containing wild-type POLD4 3′UTR (POLD4 3′UTR-wt) and miR-1200 mimics in SGC-7901/CDDP and MKN-45/CDDP cells, the luciferase reporter activities were significantly suppressed compared with transfection with POLD4 3′UTR-wt plasmid and miR-NC mimics, respectively; however, there were no significant changes of the luciferase reporter activities between cotransfection with mutant POLD4 3′UTR (POLD4 3′UTR-mt) plasmid/miR-1200 mimics and POLD4 3′UTR-mt plasmid/miR-NC mimics (Figures 4(b) and 4(c)). As determined by RT-qPCR, the mRNA levels of POLD4 decreased dramatically after transfection with miR-1200 mimics compared with control miR-NC in both SGC-7901/CDDP and MKN-45/CDDP cells (Figure 4(d)). Moreover, forced expression of miR-1200 also decreased the protein levels of POLD4 in SGC-7901/CDDP and MKN-45/CDDP cells as determined by western blot (Figure 4(e)). Therefore, POLD4 was a direct target of miR-1200 in CDDP-resistant gastric cancer cells.

### 3.5. miR-1200 Rescued the Decreased CDDP Resistance due to circ_0026359 Depletion in CDDP-Resistant Gastric Cancer Cells

**Figure 5:**
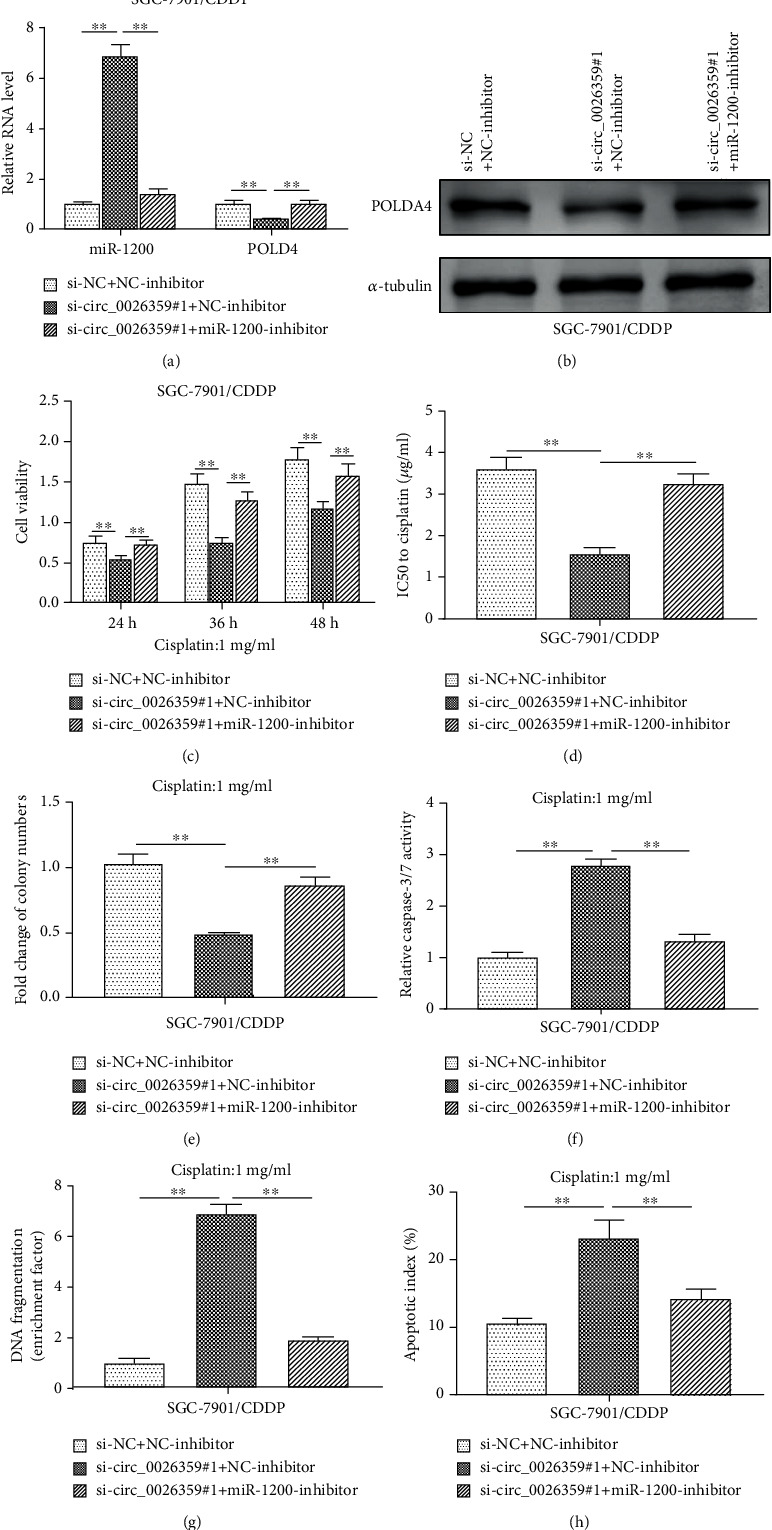
miR-1200 rescued the decreased CDDP resistance due to circ_0026359 depletion in CDDP-resistant gastric cancer cells. SGC-7901/CDDP cells were cotransfected with si-circ_0026359#1/si-NC and miR-1200 inhibitor/NC-inhibitor. (a) Expression levels of miR-1200 and POLD4 mRNA were examined by RT-qPCR. U6 and GAPDH were detected as control, respectively. (b) Protein levels of POLD4 were examined by western blot. *α*-Tubulin was detected as control. (c) MTT assay (1 *μ*g/ml CDDP treated for 24, 36, and 48 hours), (d) IC50 analysis for CDDP, (e) cell colony formation assay (1 *μ*g/ml CDDP treated for 48 hours), (f) caspase-3/7 assay (1 *μ*g/ml CDDP treated for 48 hours), (g) DNA fragmentation assay (1 *μ*g/ml CDDP treated for 48 hours), and (h) flow cytometry with Annexin V-FITC and PI staining (1 *μ*g/ml CDDP treated for 48 hours) were performed, respectively, in SGC-7901/CDDP cells after cotransfection. ^∗∗^*P* < 0.01.

For further study, combined functional experiments were performed. As shown in Figure 5(a), the mRNA levels of miR-1200 increased significantly after cotransfection with si-circ_0026359#1 and NC-inhibitor in SGC-7901/CDDP cells, but this increase was abolished by cotransfection of si-circ_0026359#1 and miR-1200-inhibitor. Concordantly, both mRNA and protein levels of POLD4 decreased significantly after cotransfection of si-circ_0026359#1 and NC-inhibitor in SGC-7901/CDDP cells, but this decrease was abolished by cotransfection of si-circ_0026359#1 and miR-1200-inhibitor (Figures 5(a) and 5(b)). The same with former results, depletion of circ_0026359 by si-circ_0026359#1 dramatically decreased cell viability on exposure to 1 *μ*g/ml CDDP during a period of 48 hours (Figure 5(c)), cell IC50 for CDDP (examined by MTT assay) (Figure 5(d)), and cell colony formation on exposure to 1 *μ*g/ml CDDP (Figure 5(e)) in SGC-7901/CDDP cells. However, these decreases were abolished by cotransfection with miR-1200-inhibitor (Figures 5(c)–5(e)). Moreover, depletion of circ_0026359 significantly promoted cell apoptosis in SGC-7901/CDDP cells treated with 1 *μ*g/ml CDDP as examined by the caspase-3/7 assay, DNA fragmentation assay, and flow cytometry, and these enhancements were abrogated by cotransfection with miR-1200-inhibitor (Figures 5(f)–5(h)). Therefore, miR-1200 rescued the decreased CDDP resistance due to circ_0026359 depletion in CDDP-resistant gastric cancer cells. The miR-1200/POLD4 pathway might mediate the role of circ_0026359 in CDDP resistance of gastric cancer cells.

## 4. Discussion

In this study, we have examined that circ_0026359 was overexpressed in human gastric cancer tissues/cells compared with normal gastric tissues/cells and was overexpressed in CDDP-resistant gastric cancer tissues/cells compared with CDDP-sensitive gastric cancer tissues/cells. Gastric cancer patients with a high level of circ_0026359 showed both low OS rates and RFS rates. On exposure to CDDP, cell proliferation decreased and cell apoptosis increased in CDDP-resistant cells SGC-7901/CDDP and MKN-45/CDDP after circ_0026359 depletion by siRNAs as determined by the MTT assay, cell colony formation assay, caspase-3/7 activity assay, and DNA fragmentation assay, respectively. Knockdown of circ_0026359 also decreased the IC50 of SGC-7901/CDDP and MKN-45/CDDP for CDDP. Therefore, we could conclude that circ_0026359 played a promoting role in CDDP resistance of gastric cancer cells. Moreover, circ_0026359 mainly located in the cytoplasm of CDDP-resistant gastric cancer cells and directly interacted with miR-1200 as determined by the luciferase reporter assay and RNA pull-down assay. In addition, POLD4 was identified to be a direct target of miR-1200 and was negatively regulated by miR-1200 in CDDP-resistant gastric cancer cells. Rescue experiments showed that miR-1200-inhibitor abrogated the decrease of cell proliferation and the increase of cell apoptosis in CDDP-resistant gastric cancer cells induced by circ_0026359 knockdown on exposure to CDDP; miR-1200-inhibitor also abolished the decrease of IC50 for CDDP induced by circ_0026359 depletion in SGC-7901/CDDP cells. As reported previously, many circRNAs (including circ_0066444, circ_0027599, and circ_0000673) played important roles in proliferation and metastasis of human gastric cancer cells [26–28]. circ_0081143 was demonstrated to promote cisplatin resistance in gastric cancer cells through targeting miR-646/CDK6 pathway [20]. circ-AKT3 promoted cisplatin resistance in gastric cancer by suppressing miR-198 and upregulating PIK3R1 [19]. We herein for the first time examined the promoting role of circ_0026359 in CDDP resistance of human gastric cancer cells.

Circular RNAs were reported to mainly act as miRNA sponges, participating in many physiological processes [29, 30]. In the present study, circ_0026359 was examined to directly interact with miR-1200 and decrease the expression levels of miR-1200 in CDDP-resistant gastric cancer cells. miR-1200-inhibitors rescued the decrease of CDDP resistance induced by depletion of circ_0026359. Therefore, miR-1200 mediated the promoting role of circ_0026359 in CDDP resistance of gastric cancer cells; miR-1200 was a suppressor in CDDP resistance of gastric cancer. As reported previously, the enhanced expression level of miR-1200 by circ-0001785 knockdown attenuated proliferative ability and induced the apoptosis of osteosarcoma cells; HOXB2 was a direct target of miR-1200 [31]. miR-1200 was also reported to be sponged by long noncoding RNA (lncRNA) RGMB-AS1 and directly target HOXB2, suppressing proliferation, migration, and invasion in glioma cells [32]. Moreover, miR-1200 was documented to be negatively correlated with the grade of tumor biology in lung neuroendocrine lung tumors [33]. These studies demonstrated that miR-1200 acted as a tumor suppressor in various kinds of human cancers. Therefore, these results were concordant with our present results in this study.

For the downstream pathway, POLD4 was examined to be a direct target of miR-1200 in CDDP-resistant gastric cancer cells. As reported, POLD4 functioned in cell proliferation and maintenance of genomic stability of human cells [34]. Low expression of POLD4 was reported to weaken the DNA repair systems including nucleotide excision repair and increase the risk of lung cancer formation [35]. Huang et al. also reported that POLD4 was low expressed in human lung cancer and was associated with poor prognosis; reduction of POLD4 in lung cancer cells resulted in cell cycle delay, checkpoint activation, and an elevated frequency of chromosomal gap/break formation [36]. In the present study, we demonstrated that depletion of circ_0026359 enhanced the activities of miR-1200 and consequently decreased the expression level of POLD4, and the CDDP resistance of gastric cancer cells decreased. Low expression levels of POLD4 might decrease the genomic stability of CDDP-resistant gastric cancer cells and consequently decrease cell viability and cell colony formation, increase caspase-3/7 activity and DNA fragmentation, and therefore decrease CDDP resistance in gastric cancer cells. In fact, in our present study, no significant change of POLD4 levels were found between gastric cancer tissues/cells and normal gastric tissues/cells, and the expression levels of POLD4 was higher in CDDP-resistant gastric cancer tissues/cells compared with CDDP-sensitive gastric cancer tissues/cells (data not shown). Therefore, the role of POLD4 showed tissue specificity in gastric cancer and lung cancer. The mechanisms might be that low levels of POLD4 disrupted genomic stability in normal lung cells and increased the risk of lung cancer formation; the formation of gastric cancer was less related with POLD4, and low levels of POLD4 disrupted genomic stability and decreased cell proliferation and CDDP resistance in gastric cancer cells. Besides POLD4, HOXB2 was also demonstrated to be a direct target of miR-1200 and mediated the tumor-suppressing role in human osteosarcoma and glioma cells [31, 32]. Therefore, miR-1200 mediated the promoting role of circ_0026359 in CDDP resistance of gastric cancer cells, and the miR-1200/POLD4 and miR-1200/HOXB2 pathways might be the downstream mechanisms involved in CDDP resistance of gastric cancer.

In summary, we have demonstrated the promoting role of circ_0026359 in CDDP resistance of human gastric cancer cells. miR-1200/POLD4 pathway was regulated by circ_0026359 and mediated the role of circ_0026359 in CDDP resistance of gastric cancer. Enhanced circ_0026359 was associated with poor survival rates in gastric cancer patients. circ_0026359 inhibitors could be used as potential adjuvant drugs in CDDP-resistant gastric cancer.

## Data Availability

The data used to support the findings of this study are available from the corresponding author upon request.

## Abbreviations

ATCC: American Type Culture Collection
CDDP: Cisplatin
circRNA: Circular RNA
mRNA: Messenger RNA
NC: Negative control
OS: Overall survival
RFS: Relapse-free survival
RT-qPCR: Reverse transcription-quantitative polymerase chain reaction
siRNA: Small interfering RNA.
